# The role of Galectin-3 in α-synuclein-induced microglial activation

**DOI:** 10.1186/s40478-014-0156-0

**Published:** 2014-11-12

**Authors:** Antonio Boza-Serrano, Juan F Reyes, Nolwen L Rey, Hakon Leffler, Luc Bousset, Ulf Nilsson, Patrik Brundin, Jose Luis Venero, Miguel Angel Burguillos, Tomas Deierborg

**Affiliations:** Experimental Neuroinflammation Laboratory, BMC, Lund University, 221 84 Lund, Sweden; Neuronal Survival Unit, BMC, Lund University, 221 84 Lund, Sweden; Translational Parkinson’s Disease Research, Center for Neurodegenerative Science, Van Andel Institute, Grand Rapids, MI USA; Department of Oncology-Pathology, Cancer Centrum Karolinska, Karolinska Institutet, 171 76 Stockholm, Sweden; Departamento de Bioquímica y Biología Molecular, Universidad de Sevilla, Facultad de Farmacia, Sevilla, Spain; Section MIG, Department of Laboratory Medicine, Solvegatan 23, Lund University, 223 62 Lund, Sweden; Centre for Analysis and Synthesis, Department of Chemistry, Lund University, PO Box 124, 221 00 Lund, Sweden; Laboratoire d’Enzymologie et Biochimie Structurales, CNRS, Bat 34, Avenue de la Terrasse, 91198 Gif-sur-Yvette, France

**Keywords:** Microglia, Galectin-3, Neuroinflammation, α-synuclein, Parkinson’s disease

## Abstract

**Background:**

Parkinson’s disease (PD) is the most prevalent neurodegenerative motor disorder. The neuropathology is characterized by intraneuronal protein aggregates of α-synuclein and progressive degeneration of dopaminergic neurons within the substantia nigra. Previous studies have shown that extracellular α-synuclein aggregates can activate microglial cells, induce inflammation and contribute to the neurodegenerative process in PD. However, the signaling pathways involved in α-synuclein-mediated microglia activation are poorly understood. Galectin-3 is a member of a carbohydrate-binding protein family involved in cell activation and inflammation. Therefore, we investigated whether galectin-3 is involved in the microglia activation triggered by α-synuclein.

**Results:**

We cultured microglial (BV2) cells and induced cell activation by addition of exogenous α-synuclein monomers or aggregates to the cell culture medium. This treatment induced a significant increase in the levels of proinflammatory mediators including the inducible Nitric Oxide Synthase (iNOS), interleukin 1 Beta (IL-1β) and Interleukin-12 (IL-12). We then reduced the levels of galectin-3 expression using siRNA or pharmacologically targeting galectin-3 activity using bis-(3-deoxy-3-(3-fluorophenyl-1*H*-1,2,3-triazol-1-yl)-β-D-galactopyranosyl)-sulfane. Both approaches led to a significant reduction in the observed inflammatory response induced by α-synuclein. We confirmed these findings using primary microglial cells obtained from wild-type and galectin-3 null mutant mice. Finally, we performed injections of α-synuclein in the olfactory bulb of wild type mice and observed that some of the α-synuclein was taken up by activated microglia that were immunopositive for galectin-3.

**Conclusions:**

We show that α-synuclein aggregates induce microglial activation and demonstrate for the first time that galectin-3 plays a significant role in microglia activation induced by α-synuclein. These results suggest that genetic down-regulation or pharmacological inhibition of galectin-3 might constitute a novel therapeutic target in PD and other synucleinopathies.

**Electronic supplementary material:**

The online version of this article (doi:10.1186/s40478-014-0156-0) contains supplementary material, which is available to authorized users.

## Introduction

Parkinson’s disease (PD) is a progressive neurodegenerative disorder clinically typified by bradykinesia, rigidity, postural instability and tremor, as well as a wide range of non-motor symptoms including constipation, bladder dysfunction and cognitive impairment [1]. Pathologically, PD is characterized by the formation of α-synuclein aggregates commonly known as Lewy bodies and Lewy neurites [2], glial activation, brain inflammation and progressive dopaminergic cell degeneration [3]. While the majority of cases of PD appear to be sporadic, genetic mutations or multiplications of the α-synuclein gene (*SNCA*) lead to the onset of familial PD [4,5].

α-Synuclein is a soluble protein composed of 140 amino acids found predominantly in presynaptic terminals where it is thought to play a role in development and plasticity [6-9]. In addition, α-synuclein is highly expressed in immune cells, including T-cells, B-cells, natural killer cells and monocytes [10]. Recent studies suggest that α-synuclein can transfer from one cell to another and promote the self-aggregation and thus possibly contributing to disease propagation [7,11-14].

While microglial activation has been suggested to play major role in the neurodegenerative process in PD [15,16], the signaling pathways that mediate this process are still poorly understood. For instance, Codolo and colleagues have recently demonstrated that α-synuclein monomers and fibrils induce Interleukin 1β (IL-1β) release from monocytes [17] via the Toll-like receptor 2 (TLR2). Moreover, Kim and colleagues have suggested that oligomeric forms of α-synuclein specifically activate TLR2 [18]. However, the TLR4 has also been implicated in α-synuclein-induced inflammation [19]. Moreover, it has been shown that the effects on cell activation and the subsequent inflammatory response can vary with the source/species of α-synuclein (mammalian cell-derived vs recombinant) and/or the type of protein used (wild type or mutant) [20]. Moreover, the molecular state of the protein used (monomeric, oligomeric or fibrillar) can also play a role in the magnitude of the inflammatory response [18]. Indeed, depending on the microenvironment/insult, activated microglia cells can adopt one of two well-characterized profiles, namely a classical (pro-inflammatory, M1) or an alternative (anti-inflammatory, M2) profile [21,22]. In these two different states, activated microglia release different factors and express different surface proteins that allow them to sense the microenvironment and coordinate the inflammatory response. In the pro-inflammatory (M1) profile, microglial cells release different pro-inflammatory molecules, *e.g.* Tumor Necrosis Factor-α (TNF-α), IL-1β, Interleukin-12 (IL-12), Interferon-γ (IFN-γ) or Nitric oxide (NO), which decrease neuronal survival [23,24]. The alternative profile, however, is characterized by release of anti-inflammatory factors (*e.g*. Interleukin-4 (IL-4), Interleukin-13 (IL-13) or Transforming Growth factor-β (TGF-β)) which reduce microglial activation [25]. While different pathways have been suggested to be involved in α-synuclein-mediated activation including the ERK 1/2, p38 MAPK, inflammasome or the NF-κβ pathway [17,26], the involvement of galectin-3 and microglial activation remains to be elucidated. Galectin-3, which is identical to the commonly used macrophage marker Mac-2, is an inflammatory mediator known to be highly expressed in some activated inflammatory cells, including microglia. Galectin-3 levels are increased in several conditions including encephalomyelitis, traumatic brain injury, experimental allergic encephalitis (EAE) and ischemic brain injury [27,28]. However, a possible role for α-synuclein induced galectin-3 activation during the inflammatory process in PD has yet to be elucidated.

Galectin-3 is a member of the β-galactoside-binding lectin family defined by their typical carbohydrate recognition domains (CRDs) [29,30]. Galectin-3 plays a role in different biological activities, including cell adhesion, proliferation, clearance, apoptosis, cell activation, cell migration, phagocytosis and inflammatory regulation [27,31-37]. Galectin-3 is found both intra- (in cytoplasm and nucleus) and extracellularly in different cell types and is suggested to play both pro-inflammatory and anti-inflammatory roles which depend on the cell type and insult provided [31,36,38,39]. In this study, we investigated whether galectin-3 is involved in microglial activation induced by α-synuclein proteins. Therefore, we exposed BV2 and primary microglia cells to monomeric and aggregated forms of recombinant α-synuclein and specifically studied the inflammatory response. We then determined the effects of microglial activation following down-regulation of galectin-3 using a specific pharmacological inhibitor or genetic down regulation using siRNA. We then monitored the effects of different forms of α-synuclein on galectin-3-null mice primary microglial cultures. Finally, we determined whether α-synuclein injections into the olfactory bulb of wild type mice result in microglia activation and galectin-3 protein expression.

## Materials and methods

### Animals

For primary microglial cultures, galectin-3 null mice [40] with a pure C57BL/6 background were obtained from Dr. K. Sävman from Gothenburg University. For intracerebral injections, 3-month-old female mice C57BL/6J were purchased from Charles River Laboratories and housed them under a 12 h light/12 h dark cycle with access to food and water and libitum at Lund university (Sweden). All procedures were carried in accordance with the international guidelines and were approved by the Malmö-Lund Ethical Committee for Animal Research in Sweden (M479-12).

### Genotyping

The genotype of gal3−/− and gal3+/+ mice was determined by an integrated extraction and amplification kit (Extract-N-Amp™, Sigma-Aldrich). The PCR consisted of 94°C for 5 min, then 40 cycles with denaturation at 94°C for 45 sec, annealing at 55°C for 30 sec, and elongation at 72°C for 1.5 min. The primers (CyberGene, Solna, Sweden) used were as follows: galectin-3 common 5-CAC GAA CGT CTT TTG CTC TCT GG-3’), gal3−/− 5-GCT TTT CTG GAT TCA TCG ACT GTG G-3’ (single band of 384 bp) and gal3+/+ 5-TGA AAT ACT TAC CGA AAA GCT GTC TGC-3’ (single band of 300 bp) [41]. We separated the PCR products by gel electrophoresis labeled with ethidium bromide and visualized in a CCD camera (SONY, Tokyo, Japan).

### Cell cultures and treatment

We cultured murine microglial cells (BV2 cell line) in Dulbecco’s modified Eagle’s medium (DMEM) containing 10% Fetal Bovine Serum (Invitrogen) with 100 U/ml Penicillin and 100 U/ml Streptomycin (Invitrogen) in 5% CO_2_ atmosphere at 37°C in T75 flasks (Nunc, Thermo Scientific) and passaged at confluency. BV2 cells were seeded at a concentration of 2×10^5^ cells/well in 24 wells plate (Nunc, Thermo Scientific) then treated with α-synuclein monomers or aggregates at different concentrations (5, 10 and 20 μM) or LPS (Sigma-Aldrich) at 1 μg/ml. All treatments were conducted for 12 h.

### Primary cell cultures

Primary microglia cultures from wild-type (WT) (C57BL/6) or galectin-3 knockout (KO) mice, cells were prepared from postnatal day 1–3 and cultured as previously described [42]. Briefly, the cerebral cortex were dissociated in ice cold Hank’s Balance Salt Solution without bivalent ions (HBSS, Invitrogen), Trypsin (0.1%) (Invitrogen) and DNase (0.05%) (Sigma-Aldrich). The cells were then plated in T75 flask with 10 ml/flask of Dulbecco’s modified Eagle’s medium (DMEM, Invitrogen) containing 10% Fetal Bovine Serum (Invitrogen) with 100 U/ml Penicillin and 100 U/ml Streptomycin (Invitrogen) in 5% CO_2_ atmosphere at 37°C. After 14 days, cells were harvested in the medium by smacking the flask 10–20 times and plated in 96 wells plates at a density of 2×10^4^ cells/well. The primary cultures were then treated with α-synuclein aggregates at different concentrations (50 nM, 200 nM, 1, 5, and 20 μM).

### α-synuclein aggregate generation

Briefly, human α-synuclein was purified using the heat treatment, ion exchange, and gel filtration chromatography as previously described [43]. α-synuclein monomers were placed on an orbital shaker at 250 rpm, shaking the monomers for 5 days at 37°C in sterile PBS. After 5 days of incubation, the protein aggregates were sonicated using a Branson Sonifier 250 (All-Spec, Willington, US) with the following conditions: 3/9 output and 30/100 Duty Cycle. We tested the composition of our aggregates and monomers using Western Blot analysis and transmission electron microscopy (TEM) (FEI, Einhofen Holland). We performed negative stain of monomeric and sonicated aggregated forms of α-synuclein by using 2% uranyl acetate in water. The concentration of endotoxin was measured in our protein preparations using the Limulus amebocyte lysate assay (Chromogenic Endotoxin Quantification Kit, Thermo Scientific, US). We detected very low levels of endotoxin (0.14 ng of LPS/ml) that was unable to influence on the microglial activation (data not shown).

### Galectin-3 inhibitor

We used a small inhibitory molecule for galectin-3 activity, bis-(3-deoxy-3-(3-fluorophenyl-1*H*-1,2,3-triazol-1-yl)-β-D-galactopyranosyl)-sulfane (K_d_ = 14 nM) [44-46] as pre-treatment 30 minutes (5, 25, 50 and 100 μM) before cells were treated with α-synuclein (monomers or aggregates) or for 12 h along with α-synuclein (monomers or aggregates) at 100 μM.

### Transfection conditions

Transfection of BV2 cells was carried out using Lipofectamine 2000 following the manufacturer’s recommendation (Life Technologies). Non-targeting control and galectin-3 siRNAs were obtained from Dharmacon. (SMART pool) siRNA sequence used: siLGal3S3(1) J-041097-09 GAGAGAUACCCAUCGCUUU, siLGal3S3(2) J-041097-10 ACUUCAAGGUUGCGGUCAA, siLGal3S3(3) J-041097-11 ACAGUGAAACCCAACGCAA, siLGal3S3(4) J-041097-12 GGAUGAAGAACCUCCGGGA.

### Western blot analysis

Briefly, proteins were loaded on 4-20% Mini-Protean TGX Precast Gels (Bio-Rad) then transferred to Nitrocellulose membranes (Bio-Rad) using Trans-Blot Turbo System (Bio-Rad). Membranes were then blocked with 10% Casein (Sigma-Aldrich) diluted in PBS (tablets, Sigma-Aldrich). After blocking, we incubated membranes, with primary antibodies at 4°C over night. We then incubated membranes with peroxidase secondary antibody (Vector Labs) and blots were developed using Clarity Western ECL Substrate (Bio-Rad) and protein levels were normalized to actin.

### Antibodies

Antibodies used for this study; anti-rabbit iNOS primary Antibody (1:5000, Santa Cruz), Anti-rat Galectin-3 Antibody (1:3000, M38 clone from Hakon Leffler’s lab), Anti-mouse Actin antibody 1:8000 (Sigma-Aldrich), Anti-human Synuclein antibody 1:3000 (Life Technologies).

### Cytokines analysis

We measured the cytokine levels from BV2 conditioned medium and primary microglial cells after 12 h treatment. We used the ultrasensitive Th1/Th2 cytokine multiplex plate to measure IFN-γ, IL-1β, IL-2, IL-4, IL-5, IL-8, IL-10, IL-12, IL-6 and TNF-α (Meso Scale Discovery, Rockville, USA) according to the manufacturer’s recommendations. The plates were analyzed using with the plate reader SECTOR Imager 6000 (Meso Scale Discovery, Rockville, USA). The conditioned medium was snap frozen on dry ice and kept in −80°C freezer prior analysis.

### Viability assay

Cell viability was performed by measuring mitochondrial activity (mitochondrial dehydrogenase) in living cells using XTT (2,3-Bis-(2-methoxy-4-nitro-5-sulfophenyl]-2H-tetrazolium-5-carboxyanilide salt) (Sigma-Aldrich). The assay was performed following manufacturer’s protocol on a 96-well plate (Biochrom Asys Expert 96 micro plate reader, Cambridge, UK).

### Olfactory bulb recombinant α-synuclein injections

We analyzed brain sections from mice injected into the olfactory bulb with different α-synuclein species (monomeric, oligomeric and fibrillar α-synuclein) as previously described [47]. Briefly, α-synuclein was produced in Escherichia coli and purified and filtered as described previously [47,48]. Oligomers were obtained by incubating soluble α-synuclein at 4 degrees for 7 days without shaking, in 50 mM Tris–HCl, and then separated from monomers by size exclusion chromatography. Fibrils were obtained from incubation of monomers under continuous shaking at 37°C, and samples were assessed by electron microscopy. α-synuclein was then tagged with ATTO-550 as described previously [47]. We injected α-synuclein monomers, oligomer and fibrils (1 mg/mL; 0.8 uL) stereotactically into the olfactory bulb of mice (coordinates AP: +5.4 mm, L: −0.75 mm, DV: −1 mm relative to bregma and dural surface). After injection, 12 h and 72 h, we perfused the mice transcardially with saline solution, followed by 4% paraformaldehyde (PFA) in phosphate buffer. We dissected the brains and post-fixed them for 2 h in PFA 4% followed by saturation in 30% sucrose solution. We then cut brains into 30 μm free-floating coronal sections, as shown previously [47].

### Immunofluorescence on mouse brain tissue

We stained free-floating coronal sections of the olfactory bulb from injected mice with primary antibodies: anti-rat Galectin-3 (1:300) and anti-rabbit Iba-1 (1:500, Wako/Nordic labs) with appropriate secondary antibodies Alexa-488 anti-rat, Alexa-647 anti-rabbit (raised in goat, 1:400, Invitrogen). We then analyzed these sections with a confocal laser microscope ZEISS LSM 510 (Switzerland), equipped with Ar and HeNe Lasers.

### Phagocytic Assay

We measured the microglial phagocytosis using a phagocytosis assay kit (Cayman Chem, USA) according to the protocol provided by the manufacturer. We plated 5× 10^4^ cells/well in 96 well plates for 12 h before treating the cells with α-synuclein (20 μM) for additional 12 h. Thereafter, IgG-FITC beads were added with or without galectin-3 inhibitor for 12 h and the phagocytic ability was then analyzed (FluoStar Optima, BMG, LabTech, Sweden).

### Statistical analysis

The differences between experimental groups were analyzed (unless otherwise stated) with one-way ANOVA with Tukey’s post hoc test, two-way ANOVA Dunnett’s post hoc test or t-test as indicated in the figure legends. P < 0.05 was considered as statistically significant. We used the statistical software GraphPad PRISM 6.0 (San Diego, CA, USA). Data is represented as mean ± S.E.M. A minimum of 3 different independent experiments were performed for all the *in vitro* experiments.

## Results

### Exogenous α-synuclein proteins promote microglial activation

**Figure 1:**
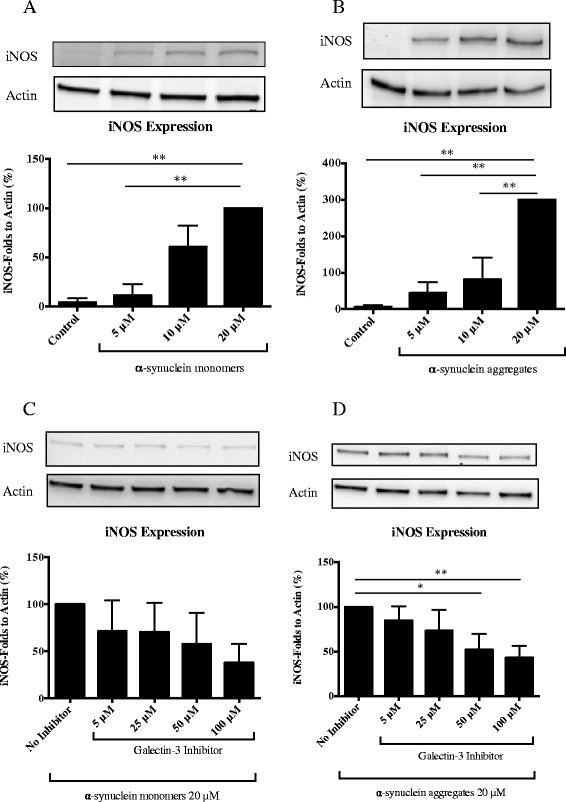
**Microglial activation by α-synuclein and inhibition by galectin-3 inhibitor.** We measured iNOS expression by western blot in microglial cells after 12 h incubation with α-synuclein monomers **(A)** and α-synuclein aggregates **(B)** using different concentrations, 5 μM, 10 μM and 20 μM. iNOS was significantly up regulated with both protein preparations of α-synuclein. α-synuclein aggregates **(B)** induced a 3-fold higher activation compared to monomers **(A)**. To determine the role of galectin-3 we used a pre-treatment, incubating the galectin-3 inhibitor for 30 min and then we incubated for 12 h the cells with α-synuclein, monomers or aggregates, using the highest concentration, 20 μM. The lower iNOS expression induced by α-synuclein monomers was not significantly inhibited by pharmacological inhibition of galectin-3 **(C)**. iNOS expression induced by α-synuclein aggregates **(D)** was inhibited by more than 50% using 100 μM of the inhibitor. We use the highest iNOS response in each experiment as an internal control to evaluate the response to the other concentrations. Western blot analysis displays iNOS and β-actin protein levels. One-way ANOVA, *P < 0.05, **P < 0.01, n = 3, mean ± S.E.M.

To assess whether α-synuclein can activate microglial cells *in vitro*, we first generated recombinant α-synuclein and induced protein aggregates as previously reported [43]. We then characterized the α-synuclein species by Western blot and electron microscopy analysis (Additional file 1: Figure S1A-C). Our data demonstrate that α-synuclein in the aggregated state is composed of a mixture of monomers, oligomers and to a lesser extent, fibrillar α-synuclein species (Additional file 1: Figure S1D). We then assessed the inflammatory response by exposing microglial cells to different concentrations of monomeric or aggregated forms of α-synuclein (5, 10 and 20 μM) for 12 h, the time period at which the temporal iNOS expression response following LPS treatment is the highest [49]. Using these conditions, we identified a concentration-dependent up-regulation of iNOS expression following both monomeric and aggregated forms of α-synuclein (Figure 1A and B, respectively). At the highest concentration used however (20 μM), α-synuclein aggregates induced a 3-fold higher iNOS expression compared to monomeric α-synuclein (Figure 1A and B). These results indicate that our α-synuclein proteins successfully induce microglial activation [17].

### Pro-inflammatory cytokine levels increase after α-synuclein treatment

**Figure 2:**
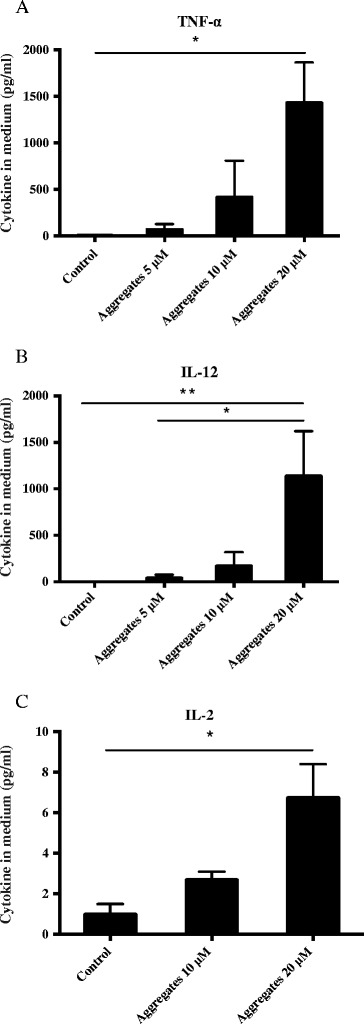
**Increased cytokine levels in BV2 microglia culture medium after α-synuclein activation.** Cytokine levels in BV2 microglia culture medium after 12 h incubation with α-synuclein aggregates at concentrations of 5, 10 and 20 μM. α-synuclein aggregates induced a significant increase in cytokine levels of the proinflammatory cytokines TNF-α **(A)**, IL-12 **(B)** and IL-2 **(C)**. One-way ANOVA, *P < 0.05, **P < 0.01, n = 3, mean ± S.E.M*.*

Following α-synuclein treatment, we observed a concentration dependent up-regulation of cytokine secretion that includes TNF-α, IL-2 and IL-12 (Figure 2A-C). These results suggest that microglial activation induced by α-synuclein aggregates promote a pro-inflammatory cascade similar to that observed in PD [24,50].

### Inhibition of galectin-3 prevents iNOS expression and reduce pro-inflammatory cytokines release in BV2 microglial cells

**Figure 3:**
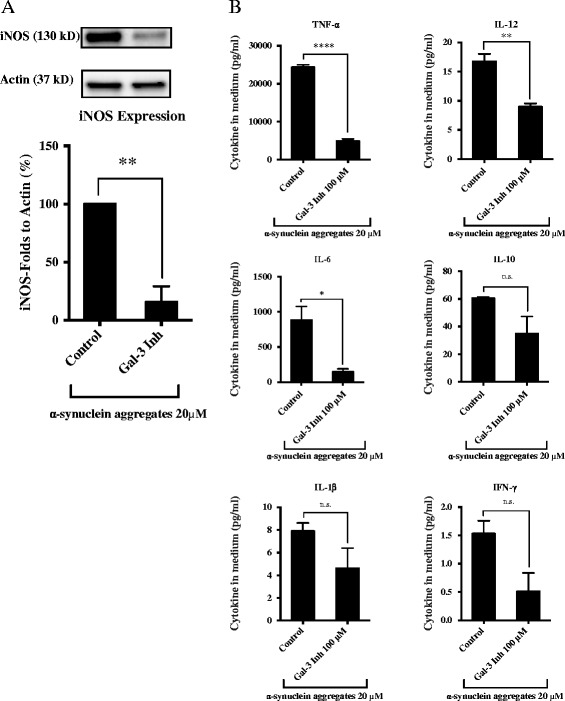
**Inhibition of microglial activation by galectin-3 inhibitor.** To determine the role of galectin-3 we used a treatment, incubating the galectin-3 inhibitor along with α-synuclein aggregates for 12 h at 20 μM. We determine by western blot the iNOS expression induced by α-synuclein aggregates. iNOS expression was inhibited by more than 80% using 100 μM of the inhibitor **(A)**. The cytokines levels were measure and TNF-α, IL-12 and IL-6 were down regulated when using the inhibitor for 12 along with α-synuclein aggregates **(B)**. We use the highest iNOS response in each experiment as an internal control to evaluate the response to the other concentrations. Western blot analysis displays iNOS and β-actin protein levels. One-way ANOVA, *P < 0,05, **P < 0.01,****P < 0,0001) n = 3, mean ± S.E.M.

First, we assessed the effect of pharmacological inhibition of galectin-3 prior to α-synuclein-induced microglial activation. To this end, microglial cells were pre-treated with a galectin-3 inhibitor for 30 minutes (5, 25, 50 and 100 μM) then washed and exposed to monomeric or α-synuclein aggregates (20 μM) then we assessed the levels of iNOS expression. After pharmacological inhibition of galectin-3, we observed a significant inhibition of α-synuclein-induced microglial activation (as shown by the lack of iNOS expression) in a concentration-dependent manner with more than 50% iNOS down-regulation following 50 and 100 μM treatment, a result that was specific to α-synuclein aggregates (Figure 1C and D). Next, we assessed the effect of pharmacological inhibition of galectin-3 for 12 h along with the α-synuclein aggregates. Pharmacological inhibition of galectin-3 for 12 h resulted in a higher inhibition (85%) of α-synuclein-induced microglial activation (iNOS expression, Figure 3A). We then measured the cytokine levels in the medium after galectin-3 inhibition and α-synuclein treatment for 12 h and observe a clear reduction in the pro-inflammatory cytokines IL-12, IL-6 and TNF-α (Figure 3B).

### Galectin-3 inhibition does not impair cell viability

As shown in figure S2, inhibition of galectin-3 does not affect cell viability when cells are treated alone or in combination with α-synuclein aggregates for 12 h. Interestingly, α-synuclein treatment of microglial cells increased mitochondrial activity with or without the inhibitor, suggesting an increased metabolic need that may be triggered by α-synuclein aggregates.

### Galectin-3 knockdown in BV2 microglial cells down-regulates iNOS expression and pro-inflammatory cytokine release

**Figure 4:**
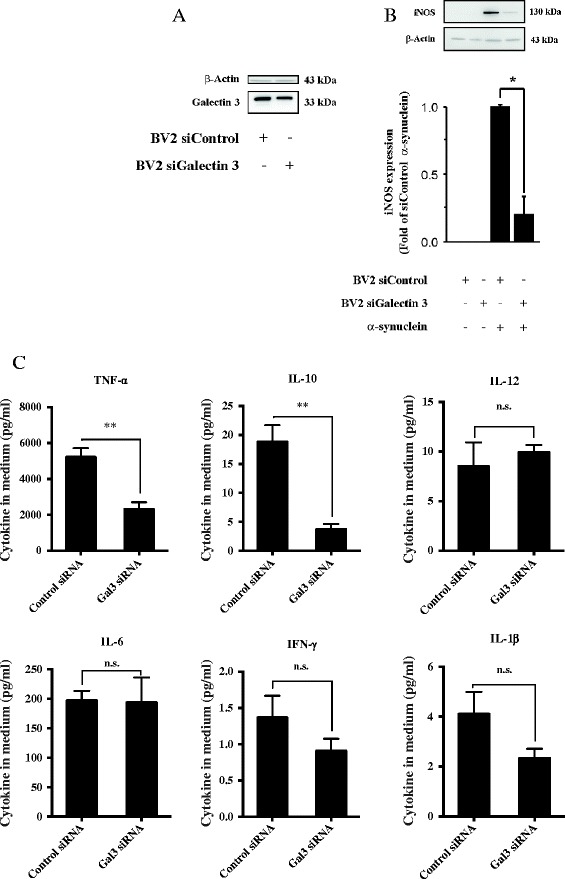
**Galectin-3 siRNA reduces microglial activation induced by α-synuclein aggregates.** BV2 microglia activated by 20 μM of α-synuclein aggregates for 12 h show a robust iNOS down regulation by 80% when galectin-3 is knocked down by siRNA **(B)**. Knock down efficiency of galectin-3 siRNA **(A)**. The cytokines levels from BV2 cells treated medium was measured after 12 h incubation with α-synuclein aggregates and we found significant reduction in TNF-α and IL-10 **(C)**. Western blot analysis showing iNOS and β-actin protein levels. t-test, One-Way ANOVA. *P < 0.05, **P < 0,01 n = 3, mean ± S.E.M.

To further test the role of galectin-3 in microglial activation, we genetically down-regulated galectin-3 expression in BV2 cells using small interfering RNA (siRNA) (Figure 4A). We then treated the cells with α-synuclein aggregates and analyzed the iNOS expression levels using Western blot analysis (Figure 3B). As expected, down-regulation of galectin-3 significantly reduced iNOS protein expression levels (Figure 4B). Next, we measured the cytokine levels in BV2 cells genetically down regulated with small interfering RNA (siRNA) targeting galectin-3 and treated with α-synuclein aggregates. Genetic down-regulation of galactin-3 also showed a reduction in TNF-α and IL-10 compared to cells treated with control siRNA (Figure 4C). Taken together these results demonstrate that down-regulation of galectin-3 reduces α-synuclein induced microglial activation and significantly lowers iNOS protein expression and cytokine up-regulation.

### Pharmacological intervention of galectin-3 reduces the microglial phagocytic activity

**Figure 5:**
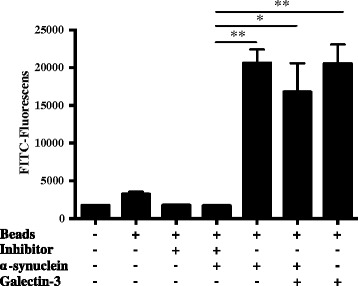
**BV2 microglial cells treated with the galectin-3 inhibitor show reduced phagocytic ability.** Phagocytic ability of microglia was robustly increased after 12 h of treatment with α-synuclein aggregates (20 μM). Adding galectin-3 inhibitor (100 μM) to microglial cultures treated with α-synuclein aggregates for the same 12 h time period robustly reduced the phagocytosis down to baseline levels. Adding galectin-3 protein we could recover the phagocytic ability even when using the inhibitor at the same time. Phagocytosis was measured by the cellular uptake up of fluorescent beads. One-way ANOVA, *P < 0.05; **P < 0.01, n = 3, mean ± S.E.M.

To test the implications on the phagocytic ability of microglial cells in our α-synuclein activation model, we treated BV2 cells with the galectin-3 inhibitor for either 30 minutes or 12h together with α-synuclein aggregates. As expected, activated microglial cells show a higher phagocytic activity whereas no differences were observed in the phagocytic ability using the inhibitor as a pre-treatment (data not shown). As shown in Figure 5, the phagocytic ability of microglia was reduced to control levels during the experiment when cells are treated with the inhibitor for 12 h. As expected, treating the cells with recombinant galectin-3 proteins up-regulates microglial phagocytic activity to levels similar to cells treated with α-synuclein aggregates (Figure 5). Importantly, we did not detect any synergic effect when cells were treated with galectin-3 and α-synuclein aggregates. These results suggest that induction of phagocytosis is an important aspect of microglial activation by α-synuclein aggregates and that galectin-3 plays an important role in cell activation and phagocytosis. These results are in in line with previous studies showing that phagocytosis is a central part in α-synuclein induced inflammation [17].

### Microglia from galectin-3 knockout mice display iNOS down-regulation following α-synuclein activation

**Figure 6:**
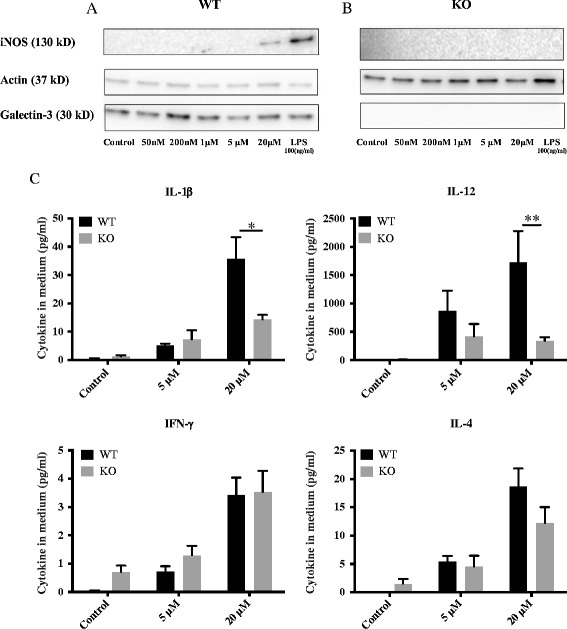
**Abrogation of iNOS proteins level and pro-inflammatory cytokines reduction in primary microglial cells from galectin-3 knockout mice after activation with α-synuclein.** Primary microglial culture from wild-type mice shows robust iNOS expression following exposure of 20 μM α-synuclein aggregates, or LPS (100 ng/ml), for 12 h **(A)**. Lower concentrations of α-synuclein aggregates, 5 μM and below, failed to induce iNOS expression in wild- type microglia **(A)**. Primary microglia from galectin-3 knockout mice completely lack iNOS up regulation following exposure of 20 μM α-synuclein aggregates for 12 h **(B)**. Cytokine levels in culture medium from primary microglial cells were measured after 12 h incubation with α-synuclein aggregates. Treatment of wild-type microglia with 5 and 20 μM α-synuclein aggregates for 12 h induced increased levels of IL-1β, IL-12, IFN-γ and IL-4 **(C)**. Treatment of galectin-3 knockout microglia for 12 h reduced levels of IL-1β IL-12 using 20 μM α-synuclein aggregates. Cytokine levels of IFN-γ and IL-4 did not change in galectin-3 knockout compared to wild-type microglia. Two-way ANOVA, *P < 0.05, **P < 0.01, n = 5, mean ± S.E.M.

Next we examined the iNOS levels in primary microglial cells, we analyzed the conditioned medium after cells been treated with α-synuclein aggregates for 12h. In line with our BV2 iNOS cytokine data (Figure 1), we identified a robust up-regulation iNOS following α-synuclein challenge (Figure 6A). Importantly, galectin-3 knockout microglial cells showed a complete abrogation of iNOS protein expression (Figure 6B). This data clearly demonstrated that iNOS regulation maybe dependent on galectin-3.

### Microglia from galectin-3 knockout mice show a down-regulation of pro-inflammatory cytokines following α-synuclein activation

To examine the cytokine levels in primary microglial cells, we analyzed the conditioned medium after cells were treated with α-synuclein aggregates. In line with our BV2 cytokine data (Figure 2), we identified a robust up-regulation of pro-inflammatory cytokines that included IL-12 and IL-1β and IFN-γ as well as the anti-inflammatory cytokine IL-4 (Figure 6C). Importantly, galectin-3 KO microglial cells showed a significant reduction in IL-1β (55%) and IL-12 (75%) cytokine release when compared to wild type microglia (Figure 6C). However, no differences were observed in IFN-γ or the anti-inflammatory cytokine IL-4. Taken together, our results indicate that galectin-3 is involved in the pro-inflammatory activation of specific inflammatory pathways that involve the IL-1β and IL-12 cytokines.

### Olfactory bulb injections of recombinant α-synuclein

**Figure 7:**
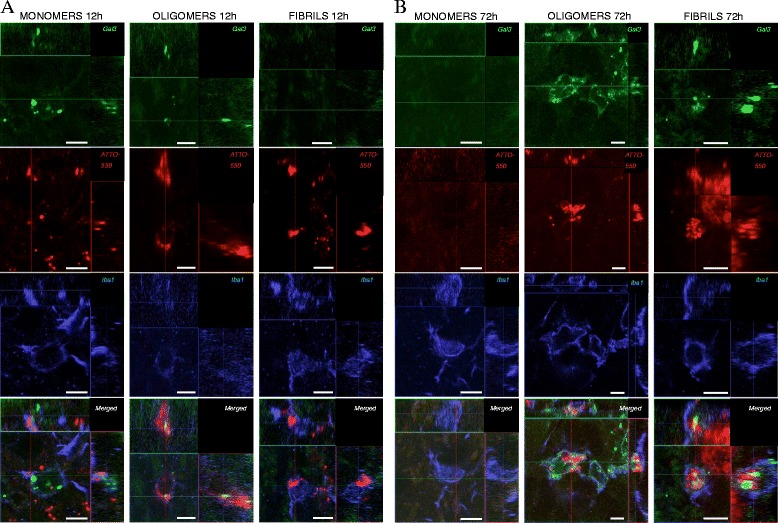
**Intracerebral injection of α-synuclein is taken up by microglia and induces galectin-3 expression.** Injections of fluorescent labeled α-synuclein (tagged with ATTO-550) into the olfactory bulbs were performed to study if α-synuclein can be taken up by microglia and induce microglial activation with galectin-3 expression. α-synuclein in the form of monomers, oligomers and fibrils were injected. Sections were stained for galectin-3 and Iba-1 (microglial markers). At 12 h post-injection, monomers and oligomers of α-synuclein were taken up by microglia and showed low expression of galectin-3 **(A)**. No microglia with galectin-3 expression was detected at 12 h after injection of fibrils. At 72 h post-injection, galectin-3 expression was clearly detected after injection of oligomers and fibrils, but had disappeared for monomers **(B)** (*n = 3*).

To confirm the expression of galectin-3 in microglial cells following activation with α-synuclein *in vivo*, we injected α-synuclein tagged with ATTO-550 in a monomeric, oligomeric or fibrillar state within the olfactory bulb of wild type mice. We then performed immunofluorescence analysis and identified activated microglial cells (Iba-1) that were positive for galectin-3 following α-synuclein injections (Figure 7). While microglial cells were able to take up all three different forms of α-synuclein injected, differences in the molecular species taken up by microglia cells were shown to vary with time. Indeed, at 12 h post injection, we identified activated microglial cells containing monomers and oligomers with up-regulated galectin-3 expression (Figure 7A). In contrast, limited galectin-3 expression was observed upon fibrillar α-synuclein (Figure 7A). These results may be due to the limited uptake of the fibrillar forms of α-synuclein [47], or the time required to phagocyte the fibrillar α-synuclein species. Interestingly, at 72 h post injection, monomeric α-synuclein did not induce galectin-3 expression, whereas oligomers and fibrils showed a clear galectin-3 up-regulation (Figure 7B). Taken together, our data demonstrate that microglial cells take up α-synuclein *in vivo* and display a microglia phenotype that is galectin-3 positive.

## Discussion

We demonstrate for the first time that galectin-3; a carbohydrate-binding protein is an immune modulator that plays an important role in the α-synuclein-induced activation of microglia. We identified a profound inflammatory inhibition of microglia cells by genetic down-regulation or pharmacological inhibition of galectin-3 or by using galectin-3 knockout primary microglia following activation by α-synuclein aggregates. In agreement with these results, prior work suggests that α-synuclein oligomers are neurotoxic and induce a strong inflammatory response in microglia cells, exceeding that seen after exposure to α-synuclein monomers [18]. Interestingly, Tokuda and colleagues have identified elevated levels of α-synuclein oligomers and an increased oligomers/total-α-synuclein ratio in the cerebrospinal fluid in PD patients, suggesting that α-synuclein oligomers may contribute to the progression of PD [51].

Recent discoveries have also demonstrated that α-synuclein can transfer from one cell to another and seed endogenous protein aggregation within the recipient cell in a prion-like fashion [13]. Besides spreading from neuron to neuron, α-synuclein can also spread from neurons to glial cells as shown previously *in vitro* and *in vivo* [52]. Due to the presence of α-synuclein in the extracellular milieu, several novel treatment strategies focusing on reducing the α-synuclein levels have been proposed including immunotherapy [53,54], delivery of α-synuclein degrading enzymes [55] or altering microglial activity [56]. Indeed, microglial activation has been linked to several neurodegenerative disorders [57] and therefore, a pharmacological intervention on the inflammatory response exerted by microglia may be a promising therapeutic target. In attempts to reduce microglial activity, several different inflammatory pathways have been targeted in earlier studies. For example peroxiredoxin 2, which inhibits the mitogen-activated protein kinase and the transcription factor nuclear factor-κB (NF-kB), have shown to be effective [58]. Additionally, minocycline, one of the most used inhibitors for microglia activation has also been suggested to specifically inhibit the M1 phenotype [59]. Moreover, inhibition of NADPH oxidase 2 (Nox2) has also been shown to reduce microglial activation in α-synuclein-induced inflammation model [60].

In this study, we used a small molecule inhibitor targeting galectin-3 and found that it inhibited microglial activation following challenge with aggregated α-synuclein. Galectin-3 inhibitor has been successfully tested in other pathological conditions with evidence for a rate-limiting role of galectin-3 [46]. For example, in a mouse model of hepatitis, the galectin-3 inhibitor attenuated liver damage and proinflammatory T cell-mediated cytokine release (IFN-γ- and IL-17- and IL-4 producing CD4+ T cells). The same inhibitor also increased the number of T cells producing the anti-inflammatory IL-10 while promoting activation of M2 phenotype in macrophages [45]. Recently, the inhibitor was shown to support the survival of pancreatic beta cells in an apoptotic model induced by proinflammatory cytokines TNF-α + IFN-γ + IL-1β [44]. In our current model system, we observed an up-regulation of both pro and anti-inflammatory cytokines released from primary and BV2 microglial cells. After analysis, we detected a significant up regulation of pro-inflammatory cytokines TNF-α, IL-2 and IL-12. Using either, the galectin-3 inhibitor for 12 h or genetic down-regulation using siRNA we found a significant down-regulation in different pro-inflammatory molecules that include iNOS and TNF-α, molecules involved in the nuclear factor-kappa Beta (NF-κβ) pathway [61]. Using primary microglial cells derived from galectin-3 knockout mice, we identified a significant reduction in IL-12 and IL-1β release compared to wild type microglia. Interestingly, the absence of galectin-3 did not significantly affect the levels of IFN-γ or cytokines related to alternative activation pathway (*e.g.* IL-4) suggesting that, in response to α-synuclein, galectin-3 plays a specific inflammatory role in microglial activation. Such selective role for galectin-3 is noteworthy as galectin-3 regulates traffic of specific membrane glycoproteins (*e.g*. receptors) [62]. While the regulatory roles of galectins vary between different cell types, this variation is likely due to the galectin type and/or the type of glycans expressed in a particular cell [63]. Our findings support the notion that the inflammatory modulation exerted by galectin-3 is related to specific inflammatory pathways.

We have identified a robust reduction of IL-12 cytokine level in the primary galectin-3 KO microglia when compared to wild type microglial cells. The IL-12 production is regulated through multiple pathways that include: NF-κβ, p38 mitogen-activated protein (MAP) kinase, cyclic adenosine monophosphate (cyclic AMP)-modulating molecules and nitric oxide (NO) [64]. In line with our findings, several studies have shown a relationship between iNOS inhibition and a down-regulation of IL-12 expression [65]. Our results demonstrate a profound iNOS expression and a pro-inflammatory cytokines reduction upon galectin-3 knockdown, gene deletion or pharmacological inhibition, suggesting that the NF-κβ pathway may indeed be the effector pathway for galectin-3. Moreover, the inflammasome, which generates mature IL-1β by activating caspase-1, has also been shown to be associated with microglial activation [66-69]. Indeed, recent findings suggest that this inflammatory signaling pathway is activated by the phagocytosis of α-synuclein [17,70]. For instance, Freeman and colleagues described a specific interaction between galectin-3 and the phagosomes/lysosomes containing α-synuclein [70]. We observed a remarkable 80% inhibition of α-synuclein-induced phagocytosis by pharmacological inhibition of galectin-3. This suggests that galectin-3 regulates α-synuclein-induced activation of microglia. On the other hand, increased phagocytosis of α-synuclein by microglia within the substantia nigra could potentially reduce the load of toxic α-synuclein species [71].

Indeed, we found galectin-3 immunoreactive microglia 12 h following injection of monomeric or oligomeric α-synuclein proteins. However, we did not detected galectin-3 immunoreactive cells after fibril injections at the same time points suggesting different up-take dynamics or intracellular processing [47]. At later time point however, α-synuclein fibrils and oligomers induced a robust galectin-3 immunoreactivity whereas monomers failed to induce a similar response indicating that monomers may be processed intracellular within 72 h without galectin-3 activation.

## Conclusions

We have demonstrated that galectin-3 is an important molecule that contributes to full-blown microglial activity upon exposure to α-synuclein aggregates. Genetic ablation, down-regulating galectin-3, or pharmacologically inhibition of galectin-3, resulted in a profound down-regulation of microglial activation (*i.e.* reduced levels of iNOS, TNF-α, IL-12, IL-1Β and the phagocytic ability of microglia). Following injections of α-synuclein species in the olfactory bulb, we observe an up-regulation of galectin-3 in microglial cells that had taken up the injected α-synuclein, providing further support for the importance of galectin-3 *in vivo*.

## Additional files

Additional file 1: Figure S1 Characterization of α-synuclein monomers and α-synuclein aggregates. We analyzed our α-synuclein preparations using Transmission Electron Micrograph (TEM) (A-C) and western blot (D). Images from TEM showed small molecules in the preparation of monomers (B) and larger molecule arrangements in our aggregated preparations (C), suggested monomeric and oligomeric/fibril proteins structures, respectively. Western Blot analysis confirmed monomeric protein in our monomer protein preparations. In our protein aggregate preparation we found oligomers and monomers and a small fraction of fibrils (>250 kDa). D_1_, normal exposure time; D_2_, long exposure time. Additional file 2: Figure S2 Survival assay showed no impairment in microglia viability after treatment with α-synuclein and/or galectin-3 inhibitor. BV2 cell viability was used to study the effect of α-synuclein aggregates and the galectin-3 inhibitor, alone or in combination after 12 h culturing. α-synuclein aggregates did not negatively affect the cell viability. In fact, α-synuclein aggregates (with or together without inhibitor) showed increased mitochondrial activity. XTT Cell Viability Assay Kit was used. One-way ANOVA, **P < 0.05, n = 4*, mean ± S.E.M.

## Abbreviations

PD: Parkinson disease
KO: Knockout
WT: Wild-type
iNOS: Inducible nitric oxide synthase
TLR: Toll like receptor
IFN-γ: Interferon gamma
TNF-α: Tumor necrosis factor alpha
IL: Interleukin
MAPK: Mitogen-activated protein kinases
ERK: Extracellular signal-regulated kinases
NF-κβ: Nuclear factor kappa-light-chain-enhancer of activated B cells
