# Supplementary figures and images for: The role of Galectin-3 in α-synuclein-induced microglial activation
Source: Acta Neuropathol Commun. 2014 Nov 12;2:156. doi: 10.1186/s40478-014-0156-0 (PMC4236422; doi:10.1186/s40478-014-0156-0)

# Supplementary Figure 1

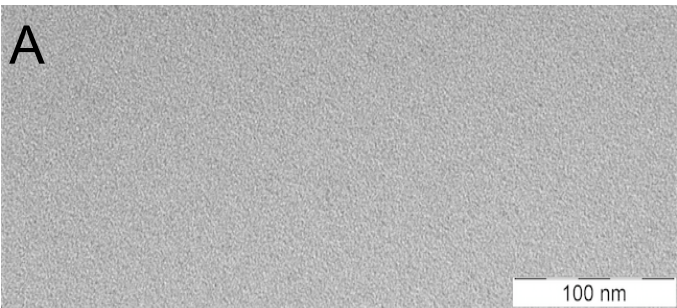

**Control**

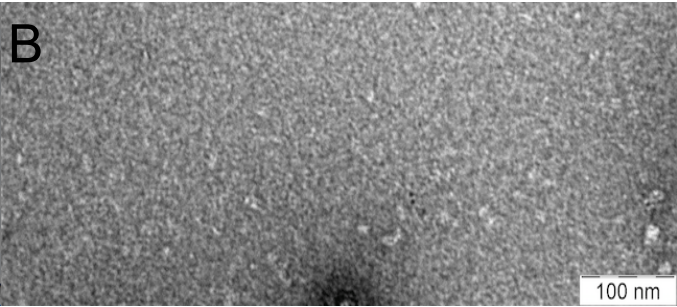

**Monomers**

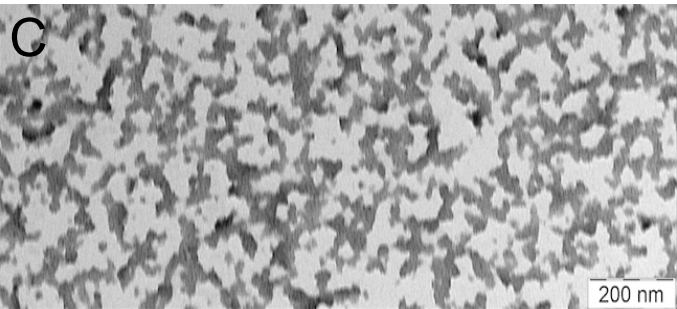

**Aggregates**

**D<sub>1</sub>**

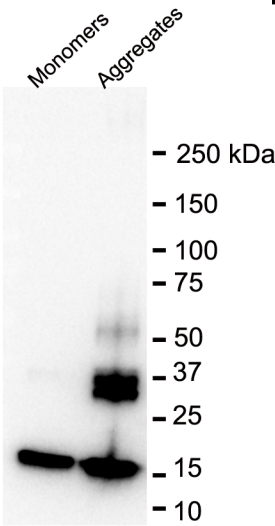

**D<sub>2</sub>**

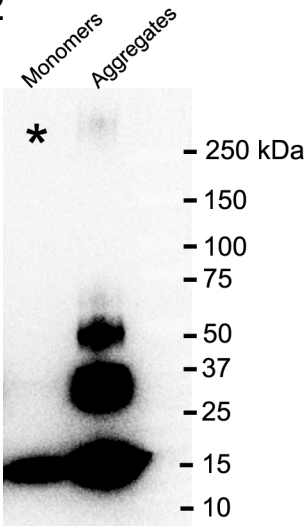

Supplement: Additional file 1: Figure S1 — Characterization of α-synuclein monomers and α-synuclein aggregates. We analyzed our α-synuclein preparations using Transmission Electron Micrograph (TEM) (A-C) and western blot (D). Images from TEM showed small molecules in the preparation of monomers (B) and larger molecule arrangements in our aggregated preparations (C), suggested monomeric and oligomeric/fibril proteins structures, respectively. Western Blot analysis confirmed monomeric protein in our monomer protein preparations. In our protein aggregate preparation we found oligomers and monomers and a small fraction of fibrils (>250 kDa). D1, normal exposure time; D2, long exposure time. [file 40478_2014_156_MOESM1_ESM.pdf]

Supplementary Figure 2

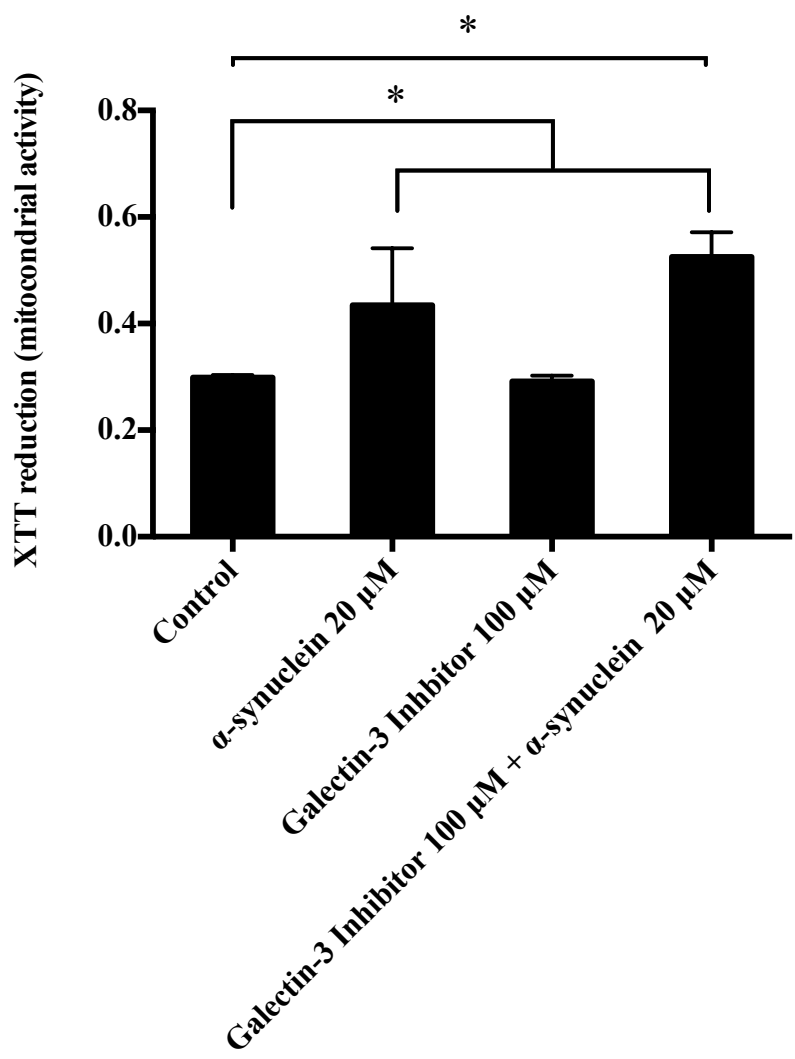

Supplement: Additional file 2: Figure S2 — Survival assay showed no impairment in microglia viability after treatment with α-synuclein and/or galectin-3 inhibitor. BV2 cell viability was used to study the effect of α-synuclein aggregates and the galectin-3 inhibitor, alone or in combination after 12 h culturing. α-synuclein aggregates did not negatively affect the cell viability. In fact, α-synuclein aggregates (with or together without inhibitor) showed increased mitochondrial activity. XTT Cell Viability Assay Kit was used. One-way ANOVA, *P < 0.05, n = 4, mean ± S.E.M. [file 40478_2014_156_MOESM2_ESM.pdf]
